# Cumulative Sonographic Features From the Morphological Uterus Sonographic Assessment (MUSA) System and Clinical Severity of Adenomyosis in Congolese Women: A Multicenter Study in Kinshasa, Democratic Republic of the Congo

**DOI:** 10.7759/cureus.118029

**Published:** 2026-10-16

**Authors:** Guy Lambert M Monzango Sibo, Ghislain Mayoka Mamoi, Stella Mpela Muzinga, Sophiane Bendifallah

**Affiliations:** 1 Gynecology, University of Kinshasa, Kinshasa, COD; 2 Obstetrics and Gynecology, University Clinics of Kinshasa, Kinshasa, COD; 3 Radiology and Medical Imaging, University Clinics of Kinshasa, Kinshasa, COD; 4 Expert Center for Endometriosis, Assistance Publique-Hôpitaux de Paris (AP-HP), Sorbonne University, Paris, FRA

**Keywords:** adenomyosis, chronic pelvic pain, dysmenorrhea, musa, sonographic phenotype, transvaginal ultrasound

## Abstract

Background: Adenomyosis is increasingly diagnosed noninvasively using transvaginal ultrasound, but the relationship between the cumulative number of standardized sonographic features and clinical severity remains incompletely understood, particularly in African populations. This study aimed to characterize adenomyosis phenotypes according to the revised Morphological Uterus Sonographic Assessment (MUSA) definitions and to evaluate the association between cumulative MUSA feature count and clinical severity.

Materials and methods: We conducted a multicenter cross-sectional observational analytical study based on retrospective review of routinely acquired clinical data and ultrasound images from three institutions in Kinshasa, Democratic Republic of the Congo. The source ultrasound examinations were performed as part of routine clinical care between January 2023 and June 16, 2026. Among 2,743 women who had undergone gynecologic ultrasonography, 211 with sonographically diagnosed adenomyosis were included. Nine revised MUSA features were assessed. The primary exposure was cumulative MUSA feature count analyzed as a continuous variable. Multivariable logistic regression models were adjusted for age, body mass index, parity, previous cesarean delivery, recorded concomitant endometriosis, uterine fibroids, and study center.

Results: The median cumulative MUSA feature count was 5 (interquartile range: 4-6). Severe chronic pelvic pain was present in 60 women (28.4%). Each additional MUSA feature was associated with increased odds of severe chronic pelvic pain (adjusted odds ratio (aOR): 1.65; 95% confidence interval (CI): 1.26-2.18; p<0.001). When direct and indirect feature counts were entered simultaneously, the indirect feature count remained associated with severe chronic pelvic pain (aOR: 1.89; 95% CI: 1.32-2.70; p<0.001), whereas the direct feature count was not statistically significant (aOR: 1.42; 95% CI: 0.97-2.07; p=0.068). Cumulative feature count was not significantly associated with severe dysmenorrhea (aOR: 1.26; 95% CI: 0.97-1.63; p=0.083) or infertility (aOR: 1.09; 95% CI: 0.87-1.38; p=0.448).

Conclusions: A higher cumulative MUSA feature count was associated with severe chronic pelvic pain, with the association mainly driven by indirect sonographic features. Cumulative MUSA feature count should be considered an exploratory phenotypic measure rather than a validated severity score. Prospective external validation using standardized image acquisition and independent blinded readers is warranted.

## Introduction

Adenomyosis is a benign uterine disorder characterized by ectopic endometrial glands and stroma within the myometrium, accompanied by reactive muscular and inflammatory changes. Its clinical expression is heterogeneous and may include dysmenorrhea, chronic pelvic pain, abnormal uterine bleeding, and reproductive dysfunction [1-3]. The development of transvaginal ultrasound and magnetic resonance imaging has shifted diagnosis from a historically histologic framework toward noninvasive evaluation, which is especially relevant for women who wish to preserve their uterus and reproductive potential [1,2,4].

To harmonize sonographic descriptions of the myometrium, the Morphological Uterus Sonographic Assessment (MUSA) group proposed standardized terminology in 2015 [5]. The revised definitions distinguish three direct features, namely, myometrial cysts, hyperechogenic islands, and echogenic subendometrial lines or buds, from six indirect features: globular uterus, asymmetrical myometrial thickening, fan-shaped shadowing, translesional vascularity, irregular junctional zone, and interrupted junctional zone [6]. The revised MUSA consensus is primarily descriptive and does not establish a universal numerical diagnostic score.

Beyond a binary diagnosis, several studies suggest that the extent or accumulation of sonographic abnormalities may relate to clinical expression. Exacoustos et al. found associations between sonographic type or extent and symptom severity [7]. Maldutyte et al. reported correlations between the number of sonographic features and both pain and menstrual blood loss [8], whereas Jha et al. described associations between several revised MUSA features and clinical symptoms [9]. However, a systematic review concluded that no single sonographic feature is currently established as a universal marker of symptom severity [10].

More recent work has explored semi-quantified approaches to sonographic severity and emphasized the need to validate reproducibility and clinical relevance [11,12]. In this context, the cumulative number of MUSA features can be investigated as an exploratory phenotyping variable without assuming that all nine features have equal biological weight or that the count constitutes a validated score.

Data from sub-Saharan Africa remain limited. Historical African series were often based on histopathology [13], and contemporary studies using standardized sonographic definitions remain uncommon. The substantial variation in reported adenomyosis frequency across clinical populations and diagnostic approaches further supports the need for locally generated data [14,15].

This study aimed to characterize the sonographic phenotypes of adenomyosis in women evaluated in Kinshasa and to assess the association between cumulative MUSA feature count and severe chronic pelvic pain. Secondary objectives were to evaluate associations with severe dysmenorrhea and infertility, compare direct and indirect feature counts, and explore potential center and diffuse-versus-focal phenotype effects.

## Materials and methods

Study design, setting, and source data period

We conducted a multicenter cross-sectional observational analytical study based on retrospective review of routinely acquired clinical data and ultrasound images from three institutions in Kinshasa, Democratic Republic of the Congo: Onyx Medical Center, the Presidential Clinic of the African Union, and the University Clinics of Kinshasa. The source ultrasound examinations included in the study had been performed between January 2023 and June 16, 2026, as part of routine clinical care and were not research-specific procedures. No prospective ultrasound examinations were performed for the purposes of this study.

At Onyx Medical Center and the Presidential Clinic of the African Union, the source transvaginal ultrasound examinations had been performed during routine clinical practice by members of the investigator team acting in their clinical roles. At the University Clinics of Kinshasa, previously acquired and archived ultrasound images were available for retrospective review. Following ethics authorization, the investigators retrospectively abstracted the relevant clinical information and reviewed the available ultrasound images according to the revised MUSA definitions. No research-specific prospective ultrasound examination or prospective research data collection occurred before ethics approval. Sonographic findings were initially interpreted by the investigator and subsequently reviewed and validated by the study supervisor; this procedure did not constitute two independent blinded readings.

Study population and operational case definition

The source dataset comprised 2,743 women who had undergone gynecologic ultrasound as part of routine care at the three participating institutions during the source data period. Women were included when retrospective review of the available clinical records and ultrasound images supported a final sonographic interpretation compatible with adenomyosis after global assessment of the myometrium and validation by the investigator and supervisor.

The revised MUSA features were used to standardize phenotyping rather than as a numerical diagnostic score; therefore, diagnosis was not based on a prespecified minimum feature count. In the final dataset, 199 of 211 cases had at least one direct MUSA feature. The 12 women without a direct feature had at least two concordant indirect features and an overall sonographic interpretation compatible with adenomyosis. A sensitivity analysis was performed after restricting the sample to women with at least one direct feature.

Examinations with incomplete imaging, insufficient image quality, or inadequate clinical information for reliable myometrial characterization were excluded.

Sonographic assessment and MUSA variables

The three direct features recorded were myometrial cysts, hyperechogenic islands, and echogenic subendometrial lines or buds. The six indirect features were globular uterus, asymmetrical myometrial thickening, fan-shaped shadowing, translesional vascularity, irregular junctional zone, and interrupted junctional zone.

Adenomyosis was additionally classified as diffuse or focal. For focal disease, the principal location was recorded as fundal, posterior, anterior, or lateral.

For each woman, the direct feature count ranged from 0 to 3, the indirect feature count from 0 to 6, and the cumulative MUSA feature count from 0 to 9. The cumulative count was treated as an exploratory sonographic phenotyping measure and was not considered a validated MUSA severity score.

Clinical variables and outcomes

Available clinical variables included age, body mass index, gravidity, parity, symptom duration, previous cesarean delivery, previous uterine curettage, previous myomectomy, and previous pregnancy loss. Recorded concomitant uterine fibroids and endometriosis were also extracted from the available clinical records.

The database did not contain a uniform protocol documenting how concomitant endometriosis had been confirmed, and it did not contain detailed fibroid characteristics such as number, size, location, or International Federation of Gynecology and Obstetrics type. These variables were therefore treated as recorded comorbidities rather than systematically ascertained conditions.

Dysmenorrhea and chronic pelvic pain intensity were recorded on a 0-10 scale. A score of at least 7 defined severe pain. Infertility was analyzed according to the information recorded in the dataset.

Hemoglobin concentration was available. Values below 10 g/dL were described as markedly low hemoglobin concentration but were not used as a surrogate for heavy menstrual bleeding and were not a primary outcome because no pictorial blood assessment chart or other standardized measure of menstrual blood loss was available.

Statistical analysis

The primary analysis treated cumulative MUSA feature count as a continuous variable. Separate multivariable logistic regression models were fitted for severe chronic pelvic pain, severe dysmenorrhea, and infertility.

Adjustment covariates were selected a priori for clinical plausibility and applied consistently across the primary models: age, body mass index, parity, previous cesarean delivery, recorded concomitant endometriosis, uterine fibroids, and study center. Study center was entered as fixed effects using indicator variables. Covariate selection was not based solely on univariable statistical significance.

Collinearity was assessed using variance inflation factors, all of which were below 1.5. No data were missing for variables included in the primary models; therefore, no imputation was performed, and all 211 women were included in each primary model.

A threshold of at least five MUSA features was not prespecified. It corresponded to the sample median and was retained only for a secondary descriptive and sensitivity analysis. The main inference therefore relied on the continuous cumulative feature count.

Exploratory analyses estimated the effects of direct and indirect feature counts separately and in a joint model containing both counts. An interaction between cumulative MUSA feature count and diffuse-versus-focal phenotype was tested. Sensitivity analyses excluded the University Clinics of Kinshasa and restricted the sample to women with at least one direct feature.

The distribution of cumulative MUSA feature count across centers was compared using the Kruskal-Wallis test. Associations are reported as adjusted odds ratios (aORs) with 95% confidence intervals (CIs). A two-sided p-value below 0.05 was considered statistically significant. Interaction and secondary analyses were interpreted as exploratory.

Analyses were performed using Python 3.13.5 (Python Software Foundation, Fredericksburg, Virginia, United States) with Statsmodels 0.14.6, SciPy 1.17.0, NumPy 2.3.5, and scikit-learn 1.8.0.

Ethics and human subjects

The study was conducted in accordance with the Declaration of Helsinki. Ethics authorization for the retrospective research review was granted by the Ethics Committee of the School of Public Health at the University of Kinshasa (approval number: ESP/CE/721/2026), with an implementation period from June 26, 2026, to June 26, 2027. All ultrasound examinations included in the present analysis had been performed before this authorization as part of routine clinical care; the last eligible ultrasound examination was performed on June 16, 2026. No ultrasound examination was performed specifically for research purposes before ethics authorization. Under the ethics authorization, the investigators conducted the retrospective review of clinical records and ultrasound images, constructed the research database, and performed the statistical analyses. Data were anonymized before analysis. Consent procedures followed the ethics committee-approved protocol.

## Results

Among 2,743 women who had undergone routine gynecologic ultrasound at the three institutions between January 2023 and June 16, 2026, 211 had sonographic findings compatible with adenomyosis on retrospective review and were included in the analysis, corresponding to 7.7% of the women in the source ultrasound population (95% CI: 6.8-8.7%). This proportion should not be interpreted as a population prevalence.

Among the 211 included women, 95 (45%) were evaluated at Onyx Medical Center, 69 (32.7%) at the Presidential Clinic of the African Union, and 47 (22.3%) at the University Clinics of Kinshasa. The mean age was 36.5±6.1 years, and the mean body mass index was 27.3±3.7 kg/m². The median parity was 2 (interquartile range (IQR): 1-3), and the median symptom duration was 2.8 years (IQR: 1.7-4.3). Previous cesarean delivery was recorded in 87 women (41.2%), uterine curettage in 68 (32.2%), myomectomy in 27 (12.8%), and previous pregnancy loss in 36 (17.1%).

Severe dysmenorrhea was present in 73 women (34.6%), severe chronic pelvic pain in 60 (28.4%), and infertility in 81 (38.4%). Thirteen women (6.2%) had hemoglobin below 10 g/dL. Recorded uterine fibroids were present in 37 women (17.5%) and recorded concomitant endometriosis in 15 (7.1%).

Sonographic phenotypes and cumulative MUSA feature count

Adenomyosis was diffuse in 159 women (75.4%) and focal in 52 (24.6%). Among focal cases, the principal location was fundal in 21 of 52 women (40.4%), posterior in 18 (34.6%), anterior in nine (17.3%), and lateral in four (7.7%).

At least one direct feature was present in 199 women (94.3%). Myometrial cysts were observed in 171 women (81%), hyperechogenic islands in 127 (60.2%), and echogenic subendometrial lines or buds in 86 (40.8%). Among indirect features, asymmetrical myometrial thickening was present in 172 women (81.5%), irregular junctional zone in 166 (78.7%), globular uterus in 140 (66.4%), interrupted junctional zone in 138 (65.4%), fan-shaped shadowing in 35 (16.6%), and translesional vascularity in 30 (14.2%).

The median direct feature count was 2 (IQR: 1-2), the median indirect feature count was 3 (IQR: 3-4), and the median cumulative MUSA feature count was 5 (IQR: 4-6). Cumulative feature count did not differ significantly across Onyx Medical Center, the Presidential Clinic of the African Union, and the University Clinics of Kinshasa, with medians of 5 (IQR: 4-6), 5 (IQR: 4-6), and 5 (IQR: 4.5-6), respectively (Kruskal-Wallis p=0.339).

Primary analysis: cumulative MUSA feature count

In the primary model adjusted for age, body mass index, parity, previous cesarean delivery, recorded concomitant endometriosis, uterine fibroids, and study center, each additional MUSA feature was associated with a 65% increase in the odds of severe chronic pelvic pain (aOR: 1.65; 95% CI: 1.26-2.18; p<0.001). For severe dysmenorrhea, the association was in the same direction but did not reach conventional statistical significance (aOR: 1.26; 95% CI: 0.97-1.63; p=0.083). No association was observed with infertility (aOR: 1.09; 95% CI: 0.87-1.38; p=0.448). Adding a quadratic term did not provide evidence of significant nonlinearity for severe chronic pelvic pain (likelihood-ratio p=0.223), which was compatible with an approximately graded linear association across the observed range.

Secondary analysis using the median threshold

A threshold of five features corresponded to the sample median and was evaluated secondarily. One hundred thirty-eight women (65.4%) had at least five features. Severe chronic pelvic pain occurred in 51 of 138 women (37%) with at least five features compared with nine of 73 women (12.3%) with fewer than five features. After adjustment using the same covariates, the threshold of at least five features was associated with severe chronic pelvic pain (aOR: 4.37; 95% CI: 1.97-9.72; p<0.001) but not with severe dysmenorrhea (aOR: 1.85; 95% CI: 0.94-3.65; p=0.075) or infertility (aOR: 1.32; 95% CI: 0.72-2.43; p=0.364) (Table 1).

**Table 1:** Association between cumulative MUSA feature count and main clinical outcomes aOR: adjusted odds ratio; CI: confidence interval; MUSA: Morphological Uterus Sonographic Assessment Models were adjusted for age, body mass index, parity, previous cesarean delivery, recorded concomitant endometriosis, uterine fibroids, and study center. The five-feature threshold corresponded to the observed sample median and was not prespecified. Statistical significance was defined as p<0.05. Individual p-values are reported in the table.

Outcome	Continuous analysis: aOR per feature	95% CI	P-value	Secondary ≥5-feature analysis: aOR	95% CI; p-value
Severe chronic pelvic pain	1.65	1.26-2.18	<0.001	4.37	1.97-9.72; <0.001
Severe dysmenorrhea	1.26	0.97-1.63	0.083	1.85	0.94-3.65; 0.075
Infertility	1.09	0.87-1.38	0.448	1.32	0.72-2.43; 0.364

Direct and indirect features and sensitivity analyses

When direct and indirect feature counts were entered simultaneously in an adjusted model, indirect feature count remained associated with severe chronic pelvic pain; each additional indirect feature was associated with an 89% increase in the odds (aOR: 1.89; 95% CI: 1.32-2.70; p<0.001). The direct feature count showed a smaller association that did not reach statistical significance (aOR: 1.42; 95% CI: 0.97-2.07; p=0.068). Neither count was significantly associated with infertility.

In exploratory adjusted models, myometrial cysts and fan-shaped shadowing remained associated with severe chronic pelvic pain when included simultaneously (myometrial cysts (aOR: 3.26; 95% CI: 1.16-9.15; p=0.025); fan-shaped shadowing (aOR: 3.44; 95% CI: 1.57-7.54; p=0.002)). Hyperechogenic islands were associated with severe dysmenorrhea (aOR: 2.68; 95% CI: 1.38-5.21; p=0.004). Previous cesarean delivery was also associated with severe dysmenorrhea in this exploratory model (aOR: 4.20; 95% CI: 2.13-8.29; p<0.001).

Diffuse adenomyosis was associated with severe dysmenorrhea after adjustment (aOR: 2.30; 95% CI: 1.04-5.10; p=0.040) but not with severe chronic pelvic pain or infertility. The interaction between cumulative MUSA feature count and diffuse-versus-focal phenotype was not significant for severe chronic pelvic pain (interaction p=0.973).

The association between cumulative MUSA feature count and severe chronic pelvic pain remained significant after exclusion of the 47 women from the University Clinics of Kinshasa, where image evaluation relied on retrospective review (n=164 (aOR: 1.64; 95% CI: 1.20-2.24; p=0.002)). It also remained significant after restriction to the 199 women with at least one direct MUSA feature (aOR: 1.55; 95% CI: 1.17-2.06; p=0.002) (Table 2).

**Table 2:** Direct and indirect feature counts and sensitivity analyses aOR: adjusted odds ratio; CI: confidence interval; MUSA: Morphological Uterus Sonographic Assessment; CUK: University Clinics of Kinshasa Joint direct/indirect models included both feature counts simultaneously and were adjusted using the same covariates as the primary model. Statistical significance was defined as p<0.05. Individual p-values are reported in the table.

Analysis	Outcome	Exposure	aOR	95% CI	P-value
Joint direct/indirect model	Severe chronic pelvic pain	Direct feature count	1.42	0.97-2.07	0.068
Joint direct/indirect model	Severe chronic pelvic pain	Indirect feature count	1.89	1.32-2.70	<0.001
Joint direct/indirect model	Severe dysmenorrhea	Direct feature count	1.39	0.96-2.02	0.080
Joint direct/indirect model	Severe dysmenorrhea	Indirect feature count	1.16	0.83-1.61	0.398
Joint direct/indirect model	Infertility	Direct feature count	1.24	0.89-1.74	0.205
Joint direct/indirect model	Infertility	Indirect feature count	0.99	0.73-1.33	0.937
Sensitivity: CUK excluded	Severe chronic pelvic pain	Total MUSA feature count	1.64	1.20-2.24	0.002
Sensitivity: ≥1 direct feature	Severe chronic pelvic pain	Total MUSA feature count	1.55	1.17-2.06	0.002

## Discussion

The principal finding of this study is that a higher cumulative number of revised MUSA features was associated with a greater likelihood of severe chronic pelvic pain among women with sonographically diagnosed adenomyosis in Kinshasa. This association remained after adjustment for clinically plausible confounders, including recorded concomitant endometriosis, uterine fibroids, and study center, and it persisted in sensitivity analyses. In contrast, cumulative feature count was not significantly associated with infertility, and its association with severe dysmenorrhea was weaker.

These findings support the concept that adenomyosis may be more informative when characterized along a sonographic phenotypic spectrum rather than as a binary present-or-absent condition. Exacoustos et al. found that sonographic type and extent were associated with symptom severity [7]. Maldutyte et al. reported correlations between the number of sonographic features and pain as well as menstrual blood loss [8]. Jha et al. also reported relationships between individual MUSA features and clinical symptoms [9]. Our study extends these observations to a Central African population and, importantly, uses the continuous feature count as the primary analysis rather than relying on a threshold selected from the observed data.

Treating feature count continuously is methodologically important. The threshold of at least five features used in a preliminary analysis corresponded to the sample median and was not prespecified; it is therefore retained only as a secondary analysis. The persistence of the association with pain when the count is modeled continuously reduces the likelihood that the conclusion is driven by a post hoc cut point. Nevertheless, the cumulative count should not be interpreted as a validated MUSA score. The nine features are not necessarily biologically independent, and simple summation implicitly assigns equal weight to each feature, which has not been established.

The separate analysis of direct and indirect components provides an additional insight. When both counts were included simultaneously, the indirect feature count remained associated with severe chronic pelvic pain, whereas the direct feature count showed only a borderline association. Indirect features reflect architectural consequences of adenomyosis involving the myometrium and junctional zone [6,10]. Our findings therefore suggest that accumulation of these architectural changes may identify a pain-related phenotype more effectively than the number of direct features alone. This interpretation remains exploratory and requires external confirmation.

At the level of individual features, myometrial cysts and fan-shaped shadowing were associated with severe chronic pelvic pain. Jha et al. also reported an association between myometrial cysts and chronic pelvic pain [9]. Hyperechogenic islands were associated with severe dysmenorrhea. However, no individual feature should be considered a definitive clinical marker, because current evidence does not support a universal sonographic predictor of symptom severity [10].

The absence of a significant association between cumulative feature count and infertility is also informative. It suggests that pain severity and reproductive dysfunction may not represent the same adenomyosis phenotype. Cumulative morphologic features may capture a pain-related phenotype more effectively than reproductive dysfunction. Reproductive outcomes may depend more strongly on specific characteristics such as subtype, location, junctional-zone involvement, or coexisting disease. A systematic review and meta-analysis showed heterogeneity in in vitro fertilization outcomes according to sonographic adenomyosis subtype, with less favorable outcomes particularly in symptomatic and diffuse disease, although certainty of evidence was low or very low for several outcomes [16].

Diffuse adenomyosis was associated with severe dysmenorrhea in our analysis, but it did not modify the association between cumulative feature count and severe chronic pelvic pain. With appropriate caution because of the smaller focal subgroup, this finding suggests that cumulative feature count may provide information that is complementary to the simple focal-versus-diffuse classification.

The association between previous cesarean delivery and severe dysmenorrhea was substantial but was not a primary hypothesis. It may reflect residual confounding related to age, parity, uterine surgery, disease phenotype, or selection. The observational design does not support a causal interpretation.

Center effects were an important concern because imaging acquisition differed between institutions. The median cumulative feature count did not differ significantly across centers, center was included in all primary models, and the association between feature count and severe chronic pelvic pain persisted after exclusion of the University Clinics of Kinshasa. These findings reduce, but do not eliminate, the possibility that the primary signal was driven by differences in imaging acquisition or review.

The operational diagnosis of adenomyosis also warrants careful interpretation. The revised MUSA consensus standardizes terminology and feature definitions but does not provide a universal numerical diagnostic rule [6]. In this study, the final diagnosis was based on global sonographic interpretation validated by the investigator and supervisor. Most women (94.3%) had at least one direct feature, whereas 12 women without a direct feature had multiple concordant indirect features. Restricting the analysis to women with at least one direct feature did not materially change the primary association. Recent efforts to grade sonographic severity similarly emphasize the need for standardization and validation rather than reliance on an unvalidated universal score [11,12].

The African setting is an additional strength of this work. The 7.7% proportion observed among women undergoing gynecologic ultrasound should not be interpreted as a population prevalence. Reported frequencies vary substantially with referral context and diagnostic method. Naftalin et al. reported adenomyosis in 20.9% of women attending a general gynecology clinic using structured transvaginal ultrasound [14], whereas an older Nigerian histopathologic series reported a different clinical-pathologic frequency [13]. Such comparisons mainly illustrate the importance of recruitment setting and diagnostic criteria.

This study has several strengths, including a multicenter population, use of revised MUSA definitions, a continuous primary exposure, explicit adjustment for clinically relevant confounders and center, absence of missing data in the primary models, formal collinearity assessment, and sensitivity analyses. Separate evaluation of direct and indirect feature counts also adds a phenotypic dimension.

Several limitations should be acknowledged. First, the study population consisted of women referred for gynecologic ultrasound, and the observed 7.7% proportion cannot be extrapolated to the general population. Second, diagnosis was not histologically confirmed in most women. Third, imaging acquisition was heterogeneous across centers, with retrospective image review at the University Clinics of Kinshasa. Fourth, investigator interpretation followed by supervisor validation was not an independent blinded reliability assessment; cumulative feature counting may amplify classification error, and future studies should use independent readers blinded to symptoms and report interobserver agreement. Fifth, systematic blinding of sonographers to clinical symptoms could not be verified, introducing potential observation bias.

Sixth, concomitant endometriosis may have been incompletely ascertained because the dataset did not document systematic dedicated endometriosis imaging; residual confounding of pain outcomes therefore cannot be excluded. Seventh, fibroids were recorded as a binary variable without detailed number, size, location, or International Federation of Gynecology and Obstetrics classification. Eighth, no pictorial blood assessment chart or other standardized measure of menstrual blood loss was available; hemoglobin below 10 g/dL should therefore not be interpreted as a surrogate for heavy menstrual bleeding. Finally, cumulative MUSA feature count is not a validated severity score and should remain an exploratory measure until externally validated.

Overall, these data support a cautious phenotyping hypothesis: a higher cumulative MUSA feature count may identify an adenomyosis phenotype associated with greater pain severity. Prospective cohorts with standardized acquisition, independent blinded readers, systematic assessment of endometriosis and fibroids, and external validation are needed before this approach can be considered for clinical use.

## Conclusions

Among Congolese women with sonographically diagnosed adenomyosis, a higher cumulative MUSA feature count was associated with severe chronic pelvic pain after adjustment for age, body mass index, parity, previous cesarean delivery, recorded concomitant endometriosis, uterine fibroids, and study center. The association was driven primarily by the number of indirect sonographic features and remained significant in sensitivity analyses.

Cumulative feature count was not significantly associated with infertility, and its association with severe dysmenorrhea was weaker. The cumulative number of MUSA features should be considered an exploratory phenotyping measure rather than a validated severity score. Prospective external validation with standardized imaging and independent blinded readers is required before clinical application.

## References

[1] Dason ES, Maxim M, Sanders A, Papillon-Smith J, Ng D, Chan C, Sobel M (2023). Guideline no. 437: diagnosis and management of adenomyosis. J Obstet Gynaecol Can.

[2] Harada T, Taniguchi F, Guo SW (2023). The Asian Society of Endometriosis and Adenomyosis guidelines for managing adenomyosis. Reprod Med Biol.

[3] Munro MG, Critchley HO, Fraser IS (2018). The two FIGO systems for normal and abnormal uterine bleeding symptoms and classification of causes of abnormal uterine bleeding in the reproductive years: 2018 revisions. Int J Gynaecol Obstet.

[4] Alcázar JL, Vara J, Usandizaga C, Ajossa S, Pascual MÁ, Guerriero S (2023). Transvaginal ultrasound versus magnetic resonance imaging for diagnosing adenomyosis: a systematic review and head-to-head meta-analysis. Int J Gynaecol Obstet.

[5] Van den Bosch T, Dueholm M, Leone FP (2015). Terms, definitions and measurements to describe sonographic features of myometrium and uterine masses: a consensus opinion from the Morphological Uterus Sonographic Assessment (MUSA) group. Ultrasound Obstet Gynecol.

[6] Harmsen MJ, Van den Bosch T, de Leeuw RA (2022). Consensus on revised definitions of Morphological Uterus Sonographic Assessment (MUSA) features of adenomyosis: results of modified Delphi procedure. Ultrasound Obstet Gynecol.

[7] Exacoustos C, Morosetti G, Conway F (2020). New sonographic classification of adenomyosis: do type and degree of adenomyosis correlate to severity of symptoms?. J Minim Invasive Gynecol.

[8] Maldutytė G, Opolskienė G, Rudaitis V, Ramašauskaitė D (2024). The association between sonographic features and clinical symptoms of adenomyosis. J Clin Ultrasound.

[9] Jha S, Prakash N, Jha VC, Sinha U (2024). New MUSA classification of adenomyosis: correlation of symptoms and clinical severity with direct and indirect features. Prz Menopauzalny.

[10] Kobayashi H, Imanaka S (2024). Understanding ultrasound features that predict symptom severity in patients with adenomyosis: a systematic review. Reprod Sci.

[11] Trommelen LM, De Leeuw RA, Van den Bosch T, Huirne JA (2025). Grading sonographic severity of adenomyosis: a pilot study assessing feasibility and interobserver reliability. J Ultrasound Med.

[12] Trommelen LM, Verbeek C, van den Bosch T, van Galen KP, Huirne JA, de Leeuw RA (2026). A novel semi-quantified method for grading sonographic severity of adenomyosis and its relation with clinical symptoms: an observational study. J Ultrasound Med.

[13] Ojo OS, Onwudiegwu U, Odesanmi WO (1991). Adenomyosis uteri in Ile-Ife, Nigeria. A prospective clinicopathologic study. Trop Geogr Med.

[14] Naftalin J, Hoo W, Pateman K, Mavrelos D, Holland T, Jurkovic D (2012). How common is adenomyosis? A prospective study of prevalence using transvaginal ultrasound in a gynaecology clinic. Hum Reprod.

[15] Mishra I, Melo P, Easter C, Sephton V, Dhillon-Smith R, Coomarasamy A (2023). Prevalence of adenomyosis in women with subfertility: systematic review and meta-analysis. Ultrasound Obstet Gynecol.

[16] Wang XL, Xu ZW, Huang YY, Lin S, Lyu GR (2023). Different subtypes of ultrasound-diagnosed adenomyosis and in vitro fertilization outcomes: a systematic review and meta-analysis. Acta Obstet Gynecol Scand.

